# Perichondritis of the auricle: bacterial or fungal? (A case series)

**DOI:** 10.1007/s00405-024-08792-w

**Published:** 2024-07-08

**Authors:** Ming Hu, Yan Cheng

**Affiliations:** 1 Department of Otorhinolaryngology Head and Neck Surgery, Tianjin First Central Hospital, Tianjin, 300192 China; 2 Institute of Otolaryngology of Tianjin, Tianjin, China; 3 Key Laboratory of Auditory Speech and Balance Medicine, Tianjin, China; 4 Key Medical Discipline of Tianjin (Otolaryngology), Tianjin, China; 5 Quality Control Centre of Otolaryngology, Tianjin, China

**Keywords:** Fungi, Metagenomic next-generation sequencing, Perichondritis, Suppurative, Treatment

## Abstract

**Background:**

Suppurative perichondritis of the auricle is a common disease that can easily cause malformations if it develops into an uncontrolled infection. In nearly half of the cases, otolaryngologists cannot identify the pathogens involved.

**Case presentation:**

In the present work, we described two cases of pyogenic perichondritis, with negative on conventional culture. However, using metagenomic next-generation sequencing (mNGS), we detected fungal infections in the patients and after the patients were given anti-fungal treatment, the patients achieved a good prognosis.

**Conclusions:**

These cases highlighted the possibility that fungi might be the involved pathogens in patients who have had multiple negative bacterial cultures, and mNGS should be applied in these cases. mNGS could be used as a supplement to traditional culture methods.

## Introduction

Suppurative perichondritis of the auricle is a cumbersome complication that occurs after traumatic damage of the auricular construction. Undoubtedly, it is imperative to tackle this disease discreetly, or it could cause the appearance of the auricle to be deformed [1]. Currently, it is recognized that *Pseudomonas aeruginosa* is the primary pathogen resulting in auricular perichondritis [2]. However, in many clinical studies, the pathogenic bacteria cultures are negative in nearly half of the cases [3]. Researchers have speculated that this may be attributed to preoperative antibiotic use or autoimmune mechanisms [4]. However, whether fungi play a vital role in these culture-negative cases has not been elucidated. Given the paucity of reports on fungal perichondritis of the auricle, we presented two cases of perichondritis of the auricle, which were diagnosed with fungal perichondritis by metagenomic next-generation sequencing (mNGS). mNGS was proposed as a promising new tool for the diagnosis of uncommon pathogens.

## Case series

### Case 1

**Fig. 1:**
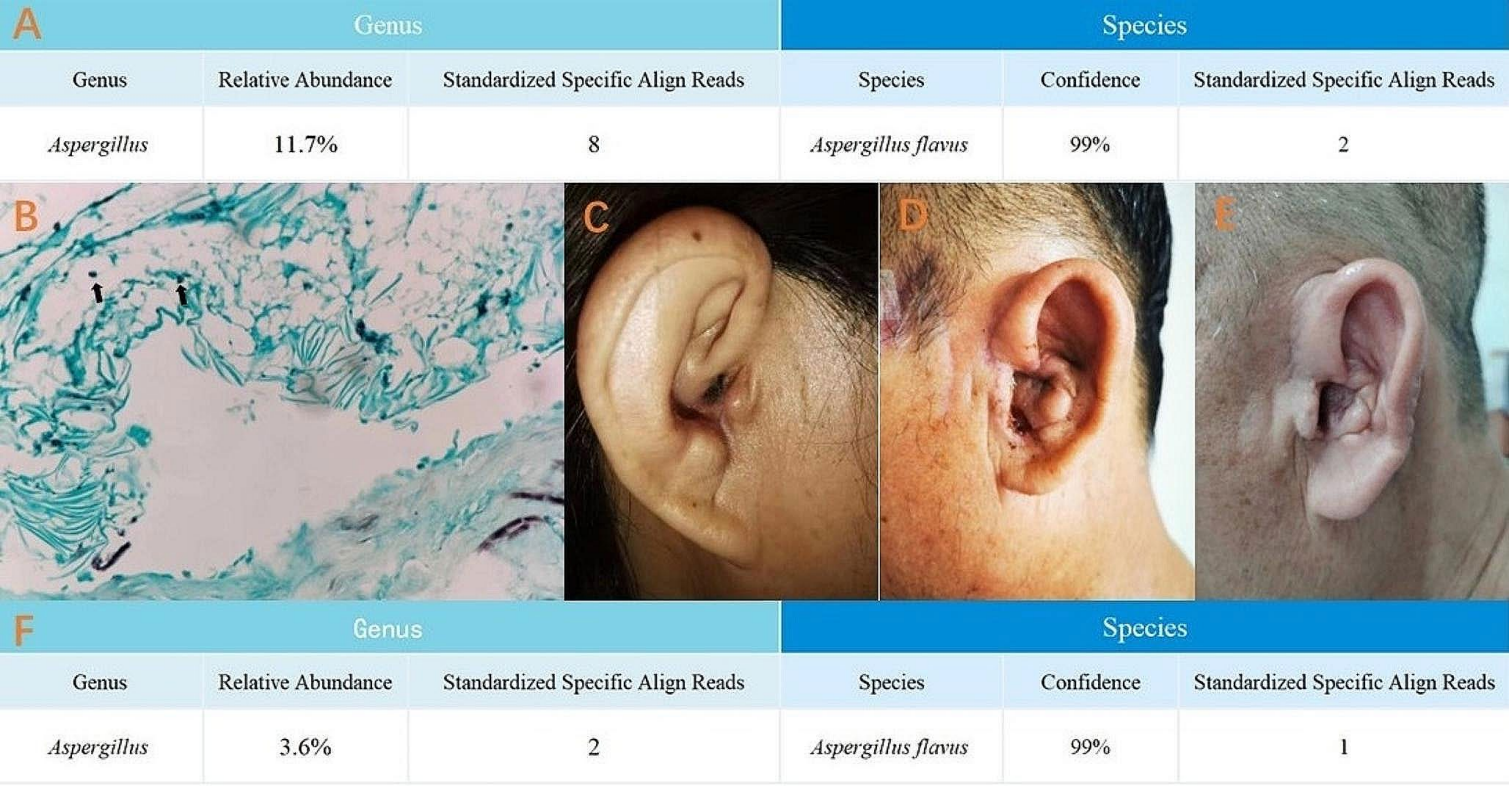
Metagenomic next-generation sequencing test results for patient 1 (**A**). GMS-stained results showed some black particles but could not find mycelia (**B**). The final result of case 1 was complete resolution of the inflammation with a minor deformity (**C**). The patient in case 2 had preoperative acute perichondritis with extensive inflammation of the auricle (**D**). Two weeks later, the external auditory canal was slightly narrow, and the local wound had healed well (**E**). Metagenomic next-generation sequencing test results for patient 2 (**F**)

A 47-year-old female had undergone a mastoidectomy 2 weeks prior, and she presented to the otolaryngology clinic with pain and suppurative effusion. She was previously in good health. Her otolaryngological examination showed a noticeable swelling of the tragus portion upon palpation. First, we treated her conservatively with antibiotics. After 7 days, the patient’s symptoms worsened, and her physical examination revealed auricle abscess formation. Debridement was executed under local anesthesia. We completely excised all of the necrotic tissues of the cartilage and perichondrium and sent the tissues for pathology. Additionally, pus was sampled for bacterial and fungal cultures and antibiotic sensitivity tests. However, no indication of a specific bacterial infection was found in the pathological examination. Multiple cultures of the pus and tissues were performed, but the results were negative. According to the literature and the pathogenic bacteria that have been proposed to be involved with cases like these, azlocillin, meropenem, and vancomycin were individually used. But the infection was not under control. We then performed a wider debridement under local anesthesia. The same disappointing situation happened, and the infection became uncontrolled. The bacterial and fungal cultures of the purulent exudate revealed no growth, and the pathology was also negative. After considering the possibility of a particular infection, mNGS was adopted. The result showed that *Aspergillus flavus* was replicated, and the sequence was 1 (Fig. 1A). GMS-stained results showed some black particles (Fig. 1B). Hence, we tentatively gave the patient voriconazole instead of antibiotics, and the patient’s symptoms were relieved gradually within two weeks. Excitingly, the patient did not develop any auricular malformations (Fig. 1C). There was no recurrence during follow-up.

### Case 2

A 53-year-old male who had a history of diabetes underwent trauma 2 months prior. Previous blood glucose control was good. He complained of swelling, pain, and suppurative effusion. The otolaryngological examination was the same as in the previous cases (Fig. 1D). We performed debridement with the same surgical approach as in case 1. Unsurprisingly, the cultures and pathology were negative again. According to the previous experiences, we applied mNGS to detect any fungal infections. The result showed that *Aspergillus flavus* was replicated, and the sequences were 2 (Fig. 1F). Then, voriconazole was prescribed, and the symptoms were relieved gradually. The patient ended up with only mild stenosis of the meatus acusticus externus, which was possibly related to the cartilage damage that had occurred at the beginning of the disease course (Fig. 1E). There was no recurrence during follow-up.

## Discussion and conclusions

In our case series, we reported two cases that presented with surgical or trauma-related perichondritis. Both had undergone multiple debridement treatments and negative pathogenic bacterial and fungal culture results throughout their disease course. To make matters worse, they experienced a failure to respond to intravenous broader spectrum antibiotic treatments that should be appropriate for common bacteria. Thankfully, these two patients were eventually confirmed to have fungal infections by mNGS and received antifungal therapy. Their infection was then controlled within two weeks, and the patients developed no obvious deformities. Therefore, we believed that fungi may also be the pathogenic bacteria of suppurative perichontitis. Our results suggested that mNGS is a powerful tool in the diagnosis of perichondritis of the auricle and has a wide range of application prospects in the differential diagnosis of ear infections.

Suppurative perichondritis always involves the perichondrium and the chondral cartilage and causes abscess formation and cavitation of these structures. Improper treatment can lead to cosmetic sequelae, especially after ear surgery, making the treatment by otolaryngologists less cosmetic [5]. At present, *Pseudomonas aeruginosa* is considered the main pathogenic bacteria, which may be related its special affinity to damaged auricular cartilage. Other suspected pathogens that have been reported include *Staphylococcus aureus* and *Escherichia coli* [5]. The treatment of perichondritis mainly relies on appropriate antibiotics, which are based on the culture results, for *Pseudomonas aeruginosa*, and the antibiotics are used in combination with debridement. The vast majority of cases recover with no sequelae. However, the infection can become uncontrollable in some cases, resulting in permanent deformities. In these cases, the patient undergoes multiple surgeries and is given multiple antibiotics throughout the disease course, but the patient still ends up with a “cauliflower ear” [6]. Some studies have revealed that antibiotic resistance and autoimmune response mechanisms may cause these uncontrolled infections [4]. However, the cause of these uncontrolled infections is frustratingly unknown.

Recently, mNGS has become an emerging method for diagnosing nonculturable microorganisms and is relatively effective in classifying and identifying pathogens, especially when the results of conventional culture are negative. mNGS is a pathogen metagenomics technology that does not rely on traditional microbial culture but rather directly extracts all nucleic acids from specimens for high-throughput sequencing. Through the analysis of biological information, the human sequence was removed, and the screened data were compared with the pathogen database to obtain the species information of suspected pathogenic microorganisms [7]. mNGS is unbiased, has wide coverage, has high sensitivity and is fast. Compared with traditional culture-based pathogen detection techniques, mNGS has the characteristics of fast detection speed, variety and high sensitivity, and has gradually become an important role in the detection of difficult and unknown pathogenic microorganisms in clinic means. However, the high cost of mNGS is not conducive to widespread promotion, and it should be used as a supplementary test for negative traditional detection methods. As a new technology for pathogen detection, mNGS has been preliminarily applied in the detection of invasive fungal sinusitis and necrotizing otitis externa in recent years, improving patient prognosis [8, 9, 10]. In our case series, both of them had multiple negative pathogenic bacterial and fungal culture results throughout their disease course. Then mNGS was used to identify the fungal infections, and good outcomes were achieved.

Collectively, we first presented a series of uncommon cases of perichondritis of the auricle. When infection occurs in patients with negative culture results, a fungal infection should be considered, and the patient and managing team should be warned of the possibility of a fungal infection. mNGS is a promising additional technique for identifying specific infections.

## References

[1] Sosin M, Weissler JM, Pulcrano M, Rodriguez ED (2015) Transcartilaginous ear piercing and infectious complications: a systematic review and critical analysis of outcomes. Laryngoscope 125(8):1827–1834. 10.1002/lary.25238Epub 2015 Mar 30 25825232 10.1002/lary.25238

[2] Klug TE, Holm N, Greve T, Ovesen T (2019) Perichondritis of the auricle: bacterial findings and clinical evaluation of different antibiotic regimens. Eur Arch Otorhinolaryngol 276(8):2199–2203. 10.1007/s00405-019-05463-zEpub 2019 May 11 31079204 10.1007/s00405-019-05463-z

[3] Forozidou E, Poutoglidis A, Tsetsos N, Kilmpasanis A, Fyrmpas G (2021) Surgery as a last resort for persistent auricular perichondritis. Ear Nose Throat J 1455613211038343. 10.1177/01455613211038343. Online ahead of print. 10.1177/01455613211038343 34375535

[4] Fang L, Xu J, Wang W, Huang Y (2020) Auricular suppurative perichondritis secondary to exclusive endoscopic ear surgery for tympanoplasty: a case report and literature review. Am J Otolaryngol 41(6):102571. 10.1016/j.amjoto.2020.102571Epub 2020 Jun 1 10.1016/j.amjoto.2020.102571 32590256

[5] Prasad HK, Sreedharan S, Prasad HS, Meyyappan MH, Harsha KS (2007) Perichondritis of the auricle and its management. J Laryngol Otol 121(6):530–534. 10.1017/S0022215107005877Epub 2007 Feb 26 17319983 10.1017/S0022215107005877

[6] Davidi E, Paz A, Duchman H, Luntz M, Potasman I (2011) Perichondritis of the auricle: analysis of 114 cases. Isr Med Assoc J 13(1):21–24 21446231

[7] Goldberg B, Sichtig H, Geyer C, Ledeboer N, Weinstock GM (2015) Making the leap from research laboratory to clinic: challenges and opportunities for next-generation sequencing in infectious disease diagnostics. mBio 6(6):e01888–e01815. 10.1128/mBio.01888-15 26646014 10.1128/mBio.01888-15 PMC4669390

[8] Wang Q, Hu R, Zhu Y, Zhu W, Jiang H (2023) Case report: the application of metagenomic next generation sequencing in diagnosing fungal malignant external otitis: a report of two cases. Front Cell Infect Microbiol 13:1236414 38053531 10.3389/fcimb.2023.1236414 PMC10694228

[9] Liang Y, Xiong X, Zhang C, Wang W, Zhang G (2022) Scedosporium apiospermum invasive rhinosinusitis in an elderly patient: diagnosis and treatment. Heliyon 8(12):e12476 36619462 10.1016/j.heliyon.2022.e12476 PMC9813714

[10] Garcia-Vidal C, Viasus D, Carratalà J (2013) Pathogenesis of invasive fungal infections. Curr Opin Infect Dis 26(3):270–276. 10.1097/QCO.0b013e32835fb920 23449139 10.1097/QCO.0b013e32835fb920

